# Machine learning predictions of unplanned readmissions using electronic medical records: Predictor importance across medical and surgical patient populations

**DOI:** 10.1371/journal.pone.0331263

**Published:** 2025-09-04

**Authors:** Michael M. Havranek, Aljoscha B. Hwang, Ilona Funke, Dominique Kuhlen, Daniel Liedtke, Stefan Boes

**Affiliations:** 1 Competence Center for Health Data Science, Faculty of Health Science and Medicine, University of Lucerne, Lucerne, Switzerland; 2 Medical Department, Hirslanden Group, Zurich, Switzerland; 3 Faculty of Health Science and Medicine, University of Lucerne, Lucerne, Switzerland; 4 Group Executive Board, Hirslanden Group, Zurich, Switzerland; Centre Hospitalier de Troyes, FRANCE

## Abstract

Hospital readmissions prolong patient suffering and increase healthcare expenditures. While several studies have attempted to develop prediction models to reduce readmissions, most have demonstrated modest predictive accuracy. To improve upon prior approaches, we conducted an overview of systematic reviews to identify the most relevant predictor variables, then subsequently developed machine learning models in a retrospective, multisite study across eight hospitals. The patient sample comprised 200,799 inpatient stays from eligible hospitalizations, based on the Centers for Medicare and Medicaid Services (CMS) definition of unplanned readmissions within 30 days of discharge. We constructed random forest models and evaluated out-of-sample performance using the area under the receiver operating characteristic curve (AUC) across different train–test splits. The hospital-wide sample was divided into medical and surgical cohorts to investigate predictor importance across different patient populations. The average AUC score was 0.78 ± 0.01 (mean ± standard deviation [SD]). Patients’ diagnoses were the most important predictor variables (contributing 18.4% ± 0.15 to the model’s decision, mean ± standard error [SE]), followed by nursing assessments (11.2% ± 0.04, mean ± SE) and procedural information (10.8% ± 0.09, mean ± SE). Comparing medical and surgical patients, we found that medications and prior healthcare use (e.g., prior emergency encounters) were more important in the medical compared with the surgical cohort, whereas procedural information and healthcare provider information (e.g., physician caseload) were more relevant in the surgical relative to the medical cohort. In conclusion, we have established the feasibility of using Swiss electronic medical record (EMR) data to accurately predict unplanned readmissions. The reported variable importances may guide future research and inform development of clinical decision support systems aimed at reducing readmissions.

## Introduction

Hospital readmissions lead to patient suffering and increase healthcare expenditures. Many countries therefore use readmission rates as quality indicators, most prominently the unplanned readmission rates within 30 days of discharge as developed and popularized by the American Centers for Medicare and Medicaid Services (CMS) [1,2]. These unplanned readmission rates have been incorporated into Switzerland’s national quality monitoring since 2022 [3]. For this reason, Swiss hospitals aiming to improve quality of care are in need of effective tools and strategies to reduce unplanned readmissions.

Multiple studies have developed prediction models for readmissions to improve quality of care and reduce healthcare expenditures, although most of the resulting models have shown modest prediction accuracy (see, e.g., [4]). The comparability of many previous studies is complicated by the fact that they use different definitions and operationalizations of readmissions (e.g., all-cause readmissions vs. condition-specific readmissions [5]). However, two studies should be mentioned in an exemplary manner in the context of the present study: The regression-based risk-adjustment models used by CMS for hospital-wide unplanned readmissions using the entire eligible US population enrolled in CMS achieved area under the receiver operating characteristic curve (AUC) values of 0.64–0.68 [6]. Another recent large-scale study from our neighboring country Germany using machine-learning models with over 4 million hospital stays attained AUC values of 0.68–0.69 [7].

The majority of previous approaches have relied on administrative data to predict readmissions. On the one hand, this has the advantage of including information that is available only after discharge (e.g., all coded diagnoses, procedures, and diagnosis-related groups [DRGs]). On the other hand, administrative data lacks other crucial information such as vital signs and medication. Electronic medical records (EMRs) contain a broader range of data sources but are limited to information available before discharge, and their use is often hindered by missing values and other challenges associated with real-world data [8,9]. In line with previous reports [10], we hypothesized that unplanned readmissions could be predicted more accurately using the EMR data available before discharge, provided that expert knowledge on predicting readmissions is combined with detailed feature engineering efforts to derive a comprehensive set of predictor variables.

To achieve this, we adopted a two-step approach. Previously, we conducted an extensive overview of systematic reviews across 440 previous studies to identify the most relevant variables for predicting unplanned readmissions (see [11] and the discussion section for more details). Now, we report the development of tree-based machine learning models to predict readmissions across hospital-wide, medical, and surgical patient populations at eight hospitals in Switzerland. As part of this second step, we invested considerable effort in feature engineering to maximize the utility of Swiss EMRs and address two central research questions: First, can unplanned readmissions within 30 days of discharge be accurately predicted using Swiss EMR data available before discharge? Second, does feature importance of predictor variables vary across different patient populations?

## Materials and methods

### Study design and data

This retrospective multisite study was conducted across eight hospitals from a leading private hospital group in Switzerland. The participating hospitals provided administrative medical data to identify unplanned readmissions using a version of the CMS algorithm adapted for the Swiss medical coding system (see below), along with medical record data to build the prediction models for the years 2018–2024.

The administrative dataset [12] contained all inpatient stays treated by the hospitals during the study period, including up to 50 diagnosis codes for each stay (from the International Statistical Classification of Diseases and Related Health Problems, 10th Revision, German Modification, ICD-10-GM [13]), up to 100 procedure codes (from the Swiss classification of surgical interventions, CHOP [13]), the DRG (from the SwissDRG system [14]), and other clinically relevant variables such as admission and discharge conditions, and patients’ demographic information.

The EMR data contained all available information from patient documentation (e.g., discharge letters, surgery reports, nursing notes, vital signs, medications, and administrative information on patients and hospitals, as well as radiology, laboratory, and other clinical results). The study was approved through a jurisdictional inquiry by the Ethics Committee Northwest & Central Switzerland (May 19, 2022; ID: Req-2022–00616). The EMR data was accessed on October 12, 2024 by an employee from the hospital group. Informed consent was not required from the patients as the researchers only received fully anonymized data that did not contain any information to identify individual patients.

### Sample, outcome, and predictors

The investigated sample consisted of 200,799 inpatient stays from eligible hospitalizations based on the CMS inclusion/exclusion criteria for a hospital-wide population (i.e., adults older than 18 years, excluding psychiatric and rehabilitation stays, etc.; see [15,16]). This hospital-wide sample was further divided into a medical cohort (n = 45,073, including cardiovascular, cardiorespiratory, neurological, and other medical patients) and a surgical/gynecologic cohort (n = 155,726; hereafter, “surgical cohort”) based on CMS definitions to build separate prediction models for these distinct patient populations.

The outcome of interest was “unplanned readmissions” within 30 days of discharge, defined as readmissions resulting from acute clinical events requiring urgent rehospitalization (i.e., not planned or foreseen during the index hospitalization [15]). Unplanned readmissions were flagged according to the CMS definitions (version 2020 [15,16]), using the CMS method as previously translated into the Swiss medical coding system and slightly modified for the Swiss healthcare setting (conceptually proposed in [17], described in [3], and validated in [18]). See S1 Appendix for a comparison between the original CMS method and our adapted version. The timeframe of 30 days was chosen because it is the most frequently employed timeframe in quality monitoring as well as prediction of unplanned readmissions [4], which has been explained by the fact that patients are particularly vulnerable to readmissions during that timeframe [19]. Only internal readmissions within the hospital group could be identified in the data from our collaborating hospitals; external readmissions outside the hospital group could not be identified within the data.

Table 1 provides a description of all predictor variables, grouped into main categories and subcategories. Selection of these variables was based on our initial overview of systematic reviews [11] and included all structured and unstructured information available in the EMRs of the collaborating hospital group. The variables include patient demographics, diagnoses, procedures, medications, prior healthcare use, admission and procedural information, vital signs, laboratory and radiology results, nursing assessments, health behaviors and living conditions, and available information on healthcare providers.

**Table 1:** Details of variables, groupings, and missing data.

Variable	Definition	Type	N cat^a^	Missing values
**Demographics**				
Age	Age in years at admission	Continuous	–	–
Sex	Sex	Categorical	2	–
Insurance	Insurance category during the hospital stay	Categorical	3	–
Foreigner	Foreign nationality/language	Binary	2	–
**Admission information**				
Admission day	Day of week of index admission	Categorical	7	–
Admission month	Month of index admission	Categorical	12	–
Admission reason	Reason for hospitalization	Categorical	4	–
Residence	Patient’s residence before index admission	Categorical	9	–
**Diagnosis-related groups**				
DRG	Working DRG at index admission [14]	Categorical	711	0.5%
**Prior healthcare use**				
Prior hospitalizations	Yes/No variable, plus number of hospitalizations within 90/180/365 days prior to index admission date	Binary/ continuous	–	–
Prior hospitalization, LOS ≥ 10 days	Yes/No variable, plus count variable of hospitalizations with LOS ≥ 10 days within 90/180/365 days prior to index admission date	Binary/ continuous	2	–
Prior ED encounters	Yes/No variable, plus number of emergency encounters within 90/180/365 days prior to index admission date	Binary/ continuous	–	–
Prior outpatient visits	Yes/No variable, plus number of outpatient visits within 90/180/365 days prior to index admission date	Binary/ continuous	–	–
**Diagnoses and comorbidities**				
All diagnoses	Known diagnoses (3-digit ICD-10 GM codes) [13]	Categorical	1,152	1.2%^c^
Condition categories	Condition category diagnosis groups that are used in risk adjustment of the unplanned readmission rates by CMS [20]	Categorical	239	–
Specific diagnosis groups	Specific diagnosis groups related to unplanned readmissions mentioned in the scientific literature (anemia, fluid and electrolyte disorders, renal failure, drug abuse, etc.) [11]	Each binary	2	–
Comorbidity count	Count of all/selected diagnoses at admission (e.g., Elixhauser groups)	Continuous	–	–
Comorbidity scores	Comorbidity scores such as the Royal College of Surgeons Charlson Comorbidity Score and Elixhauser Comorbidity Index [6,21]	Categorical/ continuous	4	–
**Procedures**				
All procedures	Performed procedures (3-digit CHOP codes) [13]	Categorical	670	3.5%^c^
Planned procedures count	Count of planned procedures at admission	Continuous	–	–
Procedure risk	Surgical procedure risk classification [22]	Categorical	6	–
**Procedural information**				
LOS	Length of stay at the day of model computation	Continuous	–	–
LEP	Patient-specific total acute care nursing services provided, in minutes [23]	Continuous	–	–
Operating room	Utilization of the operating room as a Yes/No variable, plus LOS in minutes	Binary/ continuous	2	–
Incision suture	Incisions suture time in minutes	Continuous	–	–
Anesthesia	Application of anesthesia as a Yes/No variable, plus duration in minutes	Binary/ continuous	2	–
Ventilation	Artificial ventilation provided as a Yes/No variable, plus duration in hours	Binary/ continuous	2	–
Recovery room	Utilization of the recovery room as a Yes/No variable, plus LOS in minutes	Binary/ continuous	2	–
Intermediate care unit	Utilization of intermediate care as a Yes/No variable, plus LOS in minutes	Binary/ continuous	2	–
Intensive care unit	Utilization of intensive care as a Yes/No variable, plus LOS in minutes	Binary/ continuous	2	–
ASA	ASA classification [24]	Categorical	8	–
NEMS	A score evaluating nine specific nursing services (effort) commonly provided in intensive care units [25]	Continuous	–	–
SAPS	Simplified Acute Physiology Score II [26]	Continuous	–	–
**Vital signs**				
Blood pressure	Systolic and diastolic blood pressure (most recent, mean, maximum, minimum, and median values), in mm Hg	Continuous	–	30.5%
Heart rate	Heart rate (most recent, mean, maximum, minimum, and median values), in bpm	Continuous	–	30.5%
Oxygen saturation	Oxygen saturation (most recent, mean, maximum, minimum, and median values), in %	Continuous	–	31.2%
Temperature	Temperature (most recent, mean, maximum, minimum, and median values), in degrees Celsius	Continuous	–	31.2%
**Laboratory results**				
Labs norm	Flags any laboratory results outside the normal range, including hematocrit, creatinine, urea, phosphate, potassium, C-reactive protein, calcium, lactate, alanine aminotransferase, alkaline phosphatase, aspartate aminotransferase, chloride, cystatin C, gamma-glutamyltransferase, HDL cholesterol, LDL cholesterol, total cholesterol, triglycerides, international normalized ratio, potassium, creatinase, lactate dehydrogenase, leukocytes, lithium, magnesium, methotrexate, natriuretic peptide, thrombocytes, troponin T	Each binary	2	–
Labs high	Flags any laboratory results higher than the upper normal value, for the same analyses as Labs norm	Each binary	2	–
Labs low	Flags any laboratory results below the lower normal value, for the same analyses as Labs norm	Each binary	2	–
Labs Yes/No	Flags the presence of laboratory results of any kind, for the same analyses as Labs norm	Each binary	2	–
POCT blood sugar	Point-of-care testing of blood sugar as a Yes/No variable, plus recorded values (most recent, minimum, maximum, and mean), in mmol/L	Binary/ continuous	2	9.7%^d^
**Medication**				
Medi all	Three-digit ATC classification codes of all administered medication during hospitalization, including reserve	Categorical	2	–
Medi specific	Specific administered medication (including reserve) of certain anatomical groups or alimentary system and metabolism, related to unplanned readmissions (e.g., beta-blockers, corticosteroids, drugs used in diabetes), on entry and during hospitalization	Categorical	2	–
Medi count	Count of distinct ATC codes present on entry or administered during hospitalization, including reserve	Continuous	–	–
Medi poly	Polymedication status on entry or during hospitalization. Polymedication refers to the number of administered medications >6, including reserve	Categorical	2	–
**Radiology**				
Rad measure count	Count of radiology measures (grouped by device and/or anatomical region)	Continuous	–	–
Rad urgency	Max urgency of radiology measures (grouped by device and/or anatomical region)	Continuous	–	–
**Nursing assessment**				
EPA mobility	Most recent values of movement, exhaustion, balance disorder, etc. [27]	Categorical	2–5	18.2–18.5%
EPA hygiene	Most recent values of personal hygiene and getting dressed	Categorical	4	18.2–18.5%
EPA diet	Most recent values of drinking, eating, nausea, artificial nutrition, etc.	Categorical	2–5	18.2–18.4%
EPA excretion	Most recent values of urine excretion, control of urine excretion, stool excretion, etc.	Categorical	2–4	18.2–18.4%
EPA cognition	Most recent values of vigilance, orientation, attention, fall-risk-increasing medication intake, etc.	Categorical	2–5	18.2–18.4%
EPA communication	Most recent values of hearing, vision, communication, challenging behavior, etc.	Categorical	3–5	18.2–18.4%
EPA sleep	Most recent values of falling asleep, sleep–wake rhythm	Categorical	3	18.2%
EPA respiration	Most recent values of tracheostomy, ventilation >24 h, chronic disorder of respiratory system, etc.	Categorical	2–3	18.4–18.6%
EPA pain	Most recent values of chronic pain, sadness, anxiety, etc.	Categorical	3–7	18.2%
EPA medical aids	Most recent values of medical aids with regard to hearing, vision, mobility, and nutrition.	Categorical	2–3	16.5%
EPA other	Includes pneumonia risk, fall risk, and malnutrition risk (minimum, maximum, most recent, and mode)	Categorical	2	50.9–70.6%
DOS	Yes/No variable for delirium observation screening, plus values for most recent, maximum, minimum, and mode [28]	Binary/ continuous	2	12.0%^d^
GCS	Yes/No variable for Glasgow Coma Scale score, plus values for most recent, maximum, minimum, and mode [29]	Binary/ continuous	2	3.9%^d^
NRS-M/R	Numeric Pain Rating Scale scores when moving/resting (most recent, mean, maximum, minimum, and median) [30]	Continuous	–	58.4–64.9%^d^
NRS	Yes/No variable for nutritional risk screening, plus values for most recent, maximum, minimum, and mode [31]	Binary/ continuous	2	4.4%^d^
SPI	Yes/No variable for self-care index, plus values for most recent, maximum, minimum, mean, and median [27]	Binary/ continuous	2	18.4%
Pain^b^	Pain (severe) within 36 h before discharge	Binary	2	4.2–40.6%^d^
Bleeding^b^	Bleeding (severe) within 36 h before discharge	Binary	2	5.4–16.7%^d^
Emotions^b^	Emotional distress	Binary	2	1.7%^d^
**Health behaviors and living conditions**				
BMI	Height (in cm), weight (in kg), and BMI	Continuous	–	39.4–44.8%
Noxae^b^	Describes regular consumption of alcoholic beverages, use of addictive substances, and smoking status with pack years	Binary/ continuous	2	41.1%^c^
Discharge destination	Planned discharge destination	Categorical	2–8	–
Living conditions^b^	Existing social network and available support at home	Categorical	2	3.2–11.4%^d^
**Healthcare provider information**				
Hospital name	Hospital name	Categorical	8	–
Hospital type	Federal Bureau of Statistics hospital typology [32]	Categorical	3	–
Patient mix	Hospital-specific proportion of foreign/private and semi-private/out-of-canton inpatients (2022)	Continuous	–	–
Beds & occupancy	Hospital-specific average number of hospital beds in service and bed occupancy rate (2022)	Continuous	–	–
Staff	Hospital-specific full-time nursing FTEs and FTEs per patient bed (2022)	Continuous	–	–
Inpatient volume	Hospital-specific number of hospitalizations for 90/180/365 days prior to index admission date	Continuous	–	–
Outpatient volume	Hospital-specific number of outpatients treated (2022)	Continuous	–	–
Physician caseload	Physician caseload and procedure volume performed 90/180/365 days prior to index admission date	Continuous	–	–

ASA, American Society of Anesthesiologists; ATC, anatomical therapeutic chemical; BMI, body mass index; CMS, Centers for Medicare and Medicaid Services; DRG, diagnosis-related group; DOS, delirium observation screening; ED, emergency department; EPA, outcome-oriented nursing assessment; FTE, full-time equivalent; GCS, Glasgow Coma Scale; ICD-10 GM, International Classification of Diseases, 10th Revision, German Modification; LEP, nurse activity record; LOS, length of stay; NEMS, Nine Equivalents of Nursing Manpower; NRS, nutritional risk screening; NRS-M/R, Numeric Pain Rating Scale – Moving/Resting; POCT, point-of-care testing; SAPS, Simplified Acute Physiology Score II; SPI, self-care index.

^a^In each case, the number of categories (N cat) was counted before exclusion of rare characteristics.

^b^Information was extracted from nursing notes using an LLM.

^c^Percentage of subjects without at least one diagnosis or procedure.

^d^Percentage of subjects with at least one data point.

Only information available before 9:00 AM on the day of discharge was used. Unstructured information (e.g., nursing notes) was processed using an internally employed large language model (LLM). Categorical variables were converted into binary dummy variables, while ICD-10 GM, CHOP, and anatomical therapeutic chemical (ATC) codes were restricted to three digits before transforming them into dummy variables. Binary variables with rare occurrences (<200 instances) were excluded from modeling.

Continuous variables were used both in their original form and as binary variables indicating whether a certain event or measurement occurred (e.g., number of prior hospitalizations, alongside yes/no information on whether a previous hospitalization occurred within a specific look-back period; see Table 1). For continuous variables with repeated measurements during stays (e.g., vital signs), different aggregation methods (including mean, median, maximum, minimum, and most recent values) were evaluated in a preselection step on the training data prior to modeling, as outlined below. Similarly, continuous variables assessing different time frames (e.g., prior hospitalizations within 3, 6, or 12 months) were preselected before modeling.

Missing values were imputed using the median, separately for the training and test data (see below). As shown in Table 1, certain variable groups had large quantities of missing data. Nevertheless, these variables were included during modeling to allow them to be used for patient subsets where they were available and relevant.

### Model building, evaluation, and interpretability

Models were built in Python (version 3.9.13, [33]) using random forests (i.e., tree-based machine learning algorithms) from the scikit-learn library [34]. Random forests were used because tree-based algorithms have been found to be well-suited for structured data and have shown an ability to handle missing data well (see, e.g., [35]). The data was split into training and test datasets at an 80:20 ratio. Preselection of comparable variables (e.g., variables with different aggregation methods, see above) was performed with SelectKBest from the scikit-learn library using analysis of variance (ANOVA) F-values [34]. To address class imbalance of the outcome, over- and under-sampling were evaluated for the training data but were not used during final modeling because they did not provide performance benefits on the training data. GridSearch was employed to select the number of estimators (n = 500), maximum depth (15), maximum features (“sqrt”), minimum number of samples for splitting (10), minimum number of samples for leaves (5), and the selection criterion (“Gini”) across three cross-validation folds of the training data. Modeling was repeated with 20 different train–test splits to ensure reproducibility. During each run, two models were built on the training data: a first model using all available variables, and a second (final) model selecting only the 75% of variables with the highest importance from the first run.

Model performance was evaluated on the test data using AUC values. Sensitivity and specificity were calculated using the optimal threshold identified via Youden’s J statistic [36]. Model interpretability was derived from analysis of Gini feature importances (i.e., the normalized total reduction of the evaluation criterion due to a particular feature). To demonstrate the results’ robustness independent of the employed machine learning algorithm, additional results are provided in S5 Appendix comparing the model performance of XGBoost (eXtreme Gradient Boosting) with those of the random forests. To build the XGBoost models, the same pipeline was used (see first paragraph) and GridSearch was employed again to select the number of estimators (n = 500), maximum depth (15), learning rate (0.01), subsample ratio (0.8), column sample by tree (0.8), L1 regularization (1), L2 regularization (5), and scale of positive weight (1). The code used in this study is available on GitHub (https://github.com/MMH-999/Pred_Read).

## Results

### Population characteristics

Table 2 summarizes the main characteristics of the three patient populations. As is typical for private hospitals in Switzerland, the sample includes a large number of surgical relative to medical patients (155,726 vs. 45,073), along with a majority of elective hospitalizations (79.3%) and, most importantly, a substantial proportion of privately insured patients (49.7%).

**Table 2:** Main characteristics of the included patient populations.

	Patient cohorts		
Characteristics	HWR	MED	SURG
Subjects, n	200,799	45,073	155,726
Unplanned readmissions, n (%)	7,755 (3.9%)	2,924 (6.5%)	4,831 (3.1%)
Age (years), mean ± SD	61.7 ± 17.6	69.4 ± 16.5	59.4 ± 17.3
Sex, %			
Female	51.1%	47.9%	52.1%
Male	48.9%	52.1%	47.9%
Insurance, %			
General	50.3%	47.3%	51.2%
Semi-private/private	49.7%	52.7%	48.8%
Admission type, %			
Elective	79.3%	42.7%	89.9%
Emergency	19.8%	55.6%	9.5%
Other	0.9%	1.7%	0.6%
ALOS (days), mean ± SD	4.3 ± 4.9	4.5 ± 4.5	4.2 ± 5.0
Most frequent working major diagnostic category (%)	Diseases and disorders of the musculoskeletal system and connective tissue (28.4%)	Diseases and disorders of the circulatory system (35.6%)	Diseases and disorders of the musculoskeletal system and connective tissue (33.3%)

ALOS, average length of stay; HWR, hospital-wide readmissions; MED, medical cohort; SURG, surgical cohort.

Out of 200,799 eligible hospitalizations, 7,755 (3.9%) were followed by an unplanned readmission within the hospital group. The percentage of unplanned readmissions was higher in the medical cohort than in the surgical cohort (6.5% vs. 3.1%). The average age was also higher for the medical compared with the surgical cohort (mean ± standard deviation [SD]: 69 ± 17 vs. 59 ± 17 years, respectively). In contrast, the proportion of elective admissions was significantly lower in the medical cohort (42.7% vs. 88.9%).

### Model performance

**Fig 1:**
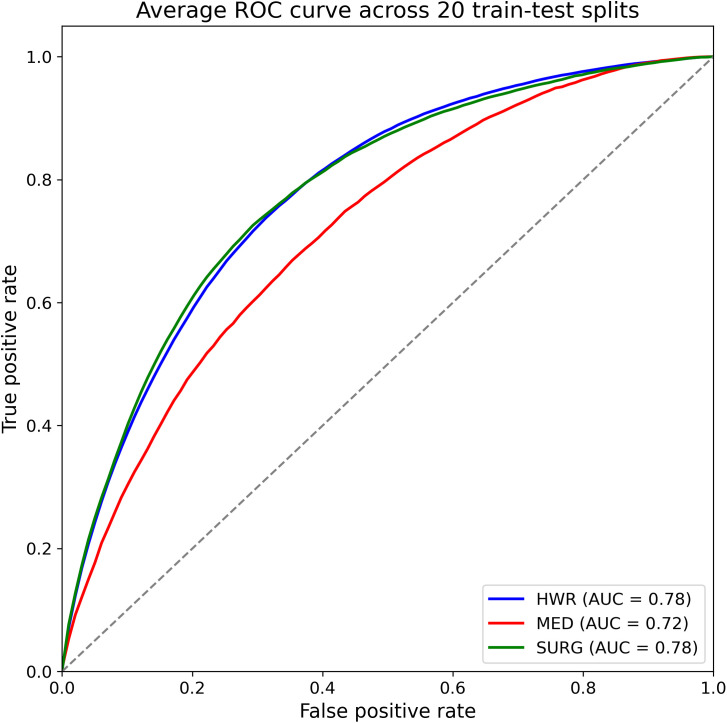
Average receiver operating characteristic (ROC) curves for the three patient populations.

Evaluation of out-of-sample model performance from the random forests was performed using test data across the different train–test splits, yielding average AUC scores of 0.78 ± 0.01 (mean ± standard deviation [SD]:) for the hospital-wide sample, 0.72 ± 0.01 for the medical cohort, and 0.78 ± 0.01 for the surgical cohort. The models yielded an average sensitivity and specificity of 0.75 ± 0.04 and 0.68 ± 0.04 for the hospital-wide sample, 0.70 ± 0.06 and 0.62 ± 0.06 for the medical cohort, 0.73 ± 0.02 and 0.71 ± 0.02 for the surgical cohort. The average model performances are visualized for the three patient populations in Fig 1. Individual performances across the various train–test splits are provided in S2–S4 Figs. In addition, the performance of the random forest models is compared with that of the XGBoost models in S5 Fig. The two machine learning algorithms yield nearly identical average AUC scores across the 20 train-test splits.

### Model interpretability

Tables 3 and 4 show the average relative feature importance for all predictor groups and subgroups across the three patient populations, based on the full set of prediction models for the 20 different train–test splits. As shown in Table 3, patients’ diagnoses are the most important predictors for unplanned readmissions (mean ± standard error [SE]: making 18.4% ± 0.15, 20.2% ± 0.14, and 17.2% ± 0.07 contribution to the model’s decision for the hospital-wide, medical, and surgical cohorts, respectively), followed by nursing assessments (11.2% ± 0.04, 12.0% ± 0.11, and 11.8% ± 0.04) and procedural information (10.8% ± 0.09, 7.7% ± 0.03, and 12.1% ± 0.03). In contrast, working DRGs (1.4% ± 0.02, 1.3% ± 0.02, 1.4% ± 0.01), patient demographics (2.5% ± 0.02, 2.6% ± 0.05, 2.5% ± 0.02), and admission information (2.6% ± 0.04, 2.6% ± 0.03, 2.3% ± 0.02) contributed least to the model’s decision.

**Table 3:** Feature importance of the main variable groups (in % ± SE).

Variable groups^a^			
	**HWR**	**MED**	**SURG**
Demographics	2.5 ± 0.02	2.6 ± 0.05	2.5 ± 0.02
Admission information	2.6 ± 0.04	2.6 ± 0.03	2.3 ± 0.02
Diagnosis-related groups	1.4 ± 0.02	1.3 ± 0.02	1.4 ± 0.01
Prior healthcare use	7.8 ± 0.04	9.4 ± 0.37	6.6 ± 0.06
Diagnoses and comorbidities	18.4 ± 0.15	20.2 ± 0.14	17.2 ± 0.07
Procedures	7.7 ± 0.02	6.4 ± 0.03	8.6 ± 0.02
Procedural information	10.8 ± 0.09	7.7 ± 0.03	12.1 ± 0.03
Vital signs	5.6 ± 0.01	6.0 ± 0.09	5.7 ± 0.01
Laboratory	8.1 ± 0.04	8.1 ± 0.04	7.6 ± 0.04
Medication	8.8 ± 0.03	9.2 ± 0.04	8.7 ± 0.02
Radiology	3.4 ± 0.02	2.8 ± 0.05	3.2 ± 0.03
Nursing assessment	11.2 ± 0.04	12.0 ± 0.11	11.8 ± 0.04
Health behaviors and living conditions	3.3 ± 0.01	3.4 ± 0.05	3.5 ± 0.01
Healthcare provider information	8.4 ± 0.04	8.3 ± 0.13	8.9 ± 0.04
**Total**	100.0	100.0	100.0

SE, standard error; HWR, hospital-wide readmissions; MED, medical cohort; SURG, surgical cohort.

^a^The color coding visualizes predictor importance. Darker green shading indicates higher importance, whereas lighter green shading indicates lower importance.

**Table 4:** Feature importance of the variable subgroups (in % ± SE).

Variable groups and subgroups^a^				
		HWR	MED	SURG
**Demographics**	Age	1.6 ± 0.02	1.6 ± 0.03	1.7 ± 0.02
	Sex	0.3 ± 0.00	0.4 ± 0.01	0.3 ± 0.00
	Insurance	0.5 ± 0.00	0.5 ± 0.01	0.5 ± 0.00
	Foreigner	0.1 ± 0.00	0.1 ± 0.00	0.1 ± 0.00
**Admission information**	Admission day	0.8 ± 0.01	0.8 ± 0.03	0.8 ± 0.01
	Admission month	1.0 ± 0.01	1.0 ± 0.02	1.0 ± 0.01
	Admission reason	0.8 ± 0.02	0.7 ± 0.03	0.5 ± 0.01
	Residence	0.1 ± 0.00	0.1 ± 0.00	0.0 ± 0.00
**Diagnosis-related groups**	DRG	1.4 ± 0.02	1.3 ± 0.02	1.4 ± 0.01
**Prior healthcare use**	Prior hospitalizations	2.4 ± 0.02	2.6 ± 0.10	2.1 ± 0.02
	Prior hospitalization, LOS ≥ 10 days	0.7 ± 0.02	1.0 ± 0.06	0.5 ± 0.01
	Prior ED encounters	3.3 ± 0.03	4.1 ± 0.22	2.7 ± 0.04
	Prior outpatient visits	1.4 ± 0.00	1.6 ± 0.01	1.4 ± 0.01
**Diagnoses and comorbidities**	All diagnoses	8.0 ± 0.12	8.6 ± 0.07	7.6 ± 0.08
	Condition categories	3.6 ± 0.03	4.2 ± 0.08	3.2 ± 0.02
	Specific diagnosis groups	2.5 ± 0.01	2.9 ± 0.01	2.2 ± 0.01
	Comorbidity count	2.9 ± 0.02	3.0 ± 0.02	2.8 ± 0.02
	Comorbidity scores	1.3 ± 0.01	1.5 ± 0.01	1.2 ± 0.01
**Procedures**	All procedures	6.0 ± 0.01	4.9 ± 0.03	6.6 ± 0.03
	Planned procedures count	1.1 ± 0.01	0.9 ± 0.02	1.4 ± 0.01
	Procedure risk	0.7 ± 0.01	0.6 ± 0.01	0.6 ± 0.01
**Procedural information**	LOS	1.4 ± 0.02	1.1 ± 0.01	1.5 ± 0.02
	LEP	2.0 ± 0.02	1.8 ± 0.03	2.3 ± 0.02
	Operating room	1.8 ± 0.02	1.5 ± 0.01	1.7 ± 0.01
	Incision suture	1.2 ± 0.01	0.8 ± 0.01	1.6 ± 0.00
	Anesthesia	1.4 ± 0.03	0.6 ± 0.02	1.5 ± 0.01
	Ventilation	0.1 ± 0.00	0.0 ± 0.00	0.1 ± 0.00
	Recovery room	0.9 ± 0.01	0.4 ± 0.00	1.1 ± 0.01
	Intermediate care unit	0.3 ± 0.00	0.2 ± 0.00	0.4 ± 0.00
	Intensive care unit	0.4 ± 0.00	0.2 ± 0.00	0.5 ± 0.01
	ASA	0.8 ± 0.02	0.5 ± 0.01	0.7 ± 0.01
	NEMS	0.3 ± 0.00	0.2 ± 0.00	0.4 ± 0.01
	SAPS	0.3 ± 0.00	0.2 ± 0.00	0.4 ± 0.01
**Vital signs**	Blood pressure	2.5 ± 0.01	2.5 ± 0.04	2.6 ± 0.01
	Heart rate	1.2 ± 0.01	1.4 ± 0.02	1.2 ± 0.00
	Oxygen saturation	0.9 ± 0.00	1.0 ± 0.01	0.9 ± 0.00
	Temperature	1.0 ± 0.00	1.2 ± 0.02	1.0 ± 0.00
**Laboratory**	Labs norm	0.9 ± 0.01	1.2 ± 0.01	0.8 ± 0.01
	Labs high	1.1 ± 0.01	1.4 ± 0.01	0.9 ± 0.01
	Labs low	0.5 ± 0.01	0.7 ± 0.01	0.4 ± 0.00
	Labs Yes/No	5.1 ± 0.07	4.4 ± 0.04	5.0 ± 0.06
	POCT blood sugar	0.4 ± 0.00	0.5 ± 0.00	0.4 ± 0.01
**Medication**	Medi all	5.3 ± 0.02	5.4 ± 0.02	5.3 ± 0.03
	Medi specific	1.2 ± 0.01	1.4 ± 0.02	1.2 ± 0.01
	Medi count	1.9 ± 0.01	2.0 ± 0.02	1.9 ± 0.01
	Medi poly	0.4 ± 0.00	0.4 ± 0.01	0.3 ± 0.00
**Radiology**	Rad measure count	1.9 ± 0.01	1.4 ± 0.03	1.8 ± 0.02
	Rad urgency	1.5 ± 0.01	1.4 ± 0.04	1.3 ± 0.02
**Nursing assessment**	EPA mobility	1.6 ± 0.01	1.6 ± 0.02	1.5 ± 0.01
	EPA hygiene	1.0 ± 0.00	1.2 ± 0.01	1.0 ± 0.00
	EPA diet	1.4 ± 0.01	1.6 ± 0.01	1.3 ± 0.01
	EPA excretion	0.8 ± 0.01	0.8 ± 0.01	0.8 ± 0.01
	EPA cognition	0.5 ± 0.01	0.5 ± 0.01	0.5 ± 0.01
	EPA communication	0.4 ± 0.01	0.5 ± 0.00	0.4 ± 0.00
	EPA sleep	0.3 ± 0.00	0.3 ± 0.01	0.3 ± 0.00
	EPA respiration	0.2 ± 0.00	0.2 ± 0.00	0.3 ± 0.00
	EPA pain	1.0 ± 0.01	1.0 ± 0.01	1.0 ± 0.01
	EPA medical aids	0.6 ± 0.00	0.6 ± 0.01	0.5 ± 0.00
	EPA other	0.4 ± 0.00	0.3 ± 0.00	0.4 ± 0.00
	DOS	0.2 ± 0.00	0.3 ± 0.00	0.2 ± 0.00
	GCS	0.1 ± 0.00	0.1 ± 0.00	0.1 ± 0.00
	NRS-M/R	0.9 ± 0.00	1.2 ± 0.02	1.3 ± 0.00
	NRS	0.1 ± 0.00	0.1 ± 0.00	0.1 ± 0.00
	SPI	1.0 ± 0.00	1.0 ± 0.02	1.2 ± 0.01
	Pain	0.5 ± 0.00	0.5 ± 0.01	0.5 ± 0.00
	Bleeding	0.2 ± 0.00	0.2 ± 0.00	0.3 ± 0.00
	Emotions	0.0 ± 0.00	0.0 ± 0.00	0.0 ± 0.00
**Health behaviors and living conditions**	BMI	2.8 ± 0.01	2.9 ± 0.05	2.9 ± 0.01
	Noxae	0.2 ± 0.00	0.1 ± 0.00	0.2 ± 0.00
	Discharge destination	0.2 ± 0.00	0.2 ± 0.00	0.2 ± 0.00
	Living conditions	0.2 ± 0.00	0.1 ± 0.00	0.2 ± 0.00
**Healthcare provider information**	Hospital name	0.6 ± 0.00	0.6 ± 0.01	0.6 ± 0.01
	Hospital type	0.1 ± 0.00	0.1 ± 0.00	0.1 ± 0.00
	Patient mix	1.3 ± 0.01	1.3 ± 0.01	1.3 ± 0.01
	Beds & occupancy	0.8 ± 0.01	0.9 ± 0.01	0.9 ± 0.01
	Staff	0.8 ± 0.01	0.8 ± 0.01	0.8 ± 0.01
	Inpatient volumne	1.5 ± 0.00	1.6 ± 0.03	1.5 ± 0.01
	Outpatient volume	0.4 ± 0.00	0.4 ± 0.01	0.4 ± 0.00
	Physician caseload	2.8 ± 0.01	2.5 ± 0.05	3.2 ± 0.01
**Total**	**Total**	100.0	100.0	100.0

SE, standard error, ASA, American Society of Anesthesiologists; ATC, anatomical therapeutic chemical; BMI, body mass index; CMS, Centers for Medicare and Medicaid Services; DRG, diagnosis-related group; DOS, delirium observation screening; ED, emergency department; EPA, outcome-oriented nursing assessment; FTE, full-time equivalent; GCS, Glasgow Coma Scale; HWR, hospital-wide readmissions; ICD-10 GM, International Classification of Diseases, 10th Revision, German Modification; LEP, nurse activity record; LOS, length of stay; MED, medical cohort; NEMS, Nine Equivalents of Nursing Manpower; NRS, nutritional risk screening; NRS-M/R, Numeric Pain Rating Scale – Moving/Resting; POCT, point-of-care testing; SAPS, Simplified Acute Physiology Score II; SPI, self-care index; SURG, surgical cohort.

^a^The color coding visualizes predictor importance. Darker green shading indicates higher importance, whereas lighter green shading indicates lower importance. Unshaded cells denote very low predictor importance (<1%).

Direct comparison of the medical and surgical patient samples showed that prior healthcare use and medications were more important in the medical than the surgical cohort (9.4% ± 0.37 vs. 6.6% ± 0.06 and 9.2% ± 0.04 vs. 8.7% ± 0.02, respectively). Conversely, procedural information and healthcare provider information were more relevant in the surgical compared with the medical cohort (12.1% ± 0.03 vs. 7.7% ± 0.03 and 8.9% ± 0.04 vs. 8.3% ± 0.13, respectively).

Looking more closely at the predictor subgroups in Table 4, the most important variables for prediction were all diagnoses, all procedures, and all medications (i.e., Medi all) used as dummy variables from ICD-10 GM (8.0% ± 0.12, 8.6% ± 0.07, and 7.6% ± 0.08 for the hospital-wide, medical, and surgical cohorts, respectively), CHOP (6.0% ± 0.01, 4.9% ± 0.03, and 6.6% ± 0.03), and ATC codes (5.3% ± 0.02, 5.4% ± 0.02, and 5.3% ± 0.03). These were followed in importance by whether a specific laboratory test was conducted during the stay (i.e., Labs Yes/No; 5.1% ± 0.07, 4.4% ± 0.04, and 5.0% ± 0.06).

Looking at the single ICD-10 GM, CHOP, and ATC codes revealed that I25 (i.e., chronic ischemic heart disease: 0.3% ± 0.01), I10 (i.e., essential (primary) hypertension: 0.3% ± 0.00), and Z95 (i.e., presence of cardiac or vascular implants or transplants: 0.2% ± 0.00) were the most important individual diagnosis codes in terms of feature importance in the hospital-wide sample. 885 (i.e., angiocardiography with contrast medium: 0.3% ± 0.01), 992 (i.e., injection or infusion of another therapeutic or prophylactic substance: 0.2% ± 0.00), and 009 (i.e., other measures and interventions: 0.2% ± 0.00) were the most relevant procedure codes and C03C (i.e., high-ceiling diuretics: 0.2% ± 0.00), C07A (i.e., beta blocking agents: 0.2% ± 0.00), and A06A (i.e., drugs for constipation: 0.2% ± 0.00) were the most important medication codes.

Considering other important variable subgroups, age was the most important predictor within the demographics group (1.6% ± 0.02, 1.6% ± 0.03, and 1.7% ± 0.02), while prior emergency encounters were most relevant factor within prior healthcare use (i.e., prior ED encounters; 3.3% ± 0.03, 4.1% ± 0.22, and 2.7% ± 0.04). Furthermore, provided nursing services was most important within procedural information (i.e., LEP; 2.0% ± 0.02, 1.8% ± 0.03, and 2.3% ± 0.02), blood pressure within vital signs (2.5% ± 0.01, 2.5% ± 0.04, and 2.6% ± 0.01), the mobility dimension within nursing assessment (i.e., EPA mobility; 1.6% ± 0.01, 1.6% ± 0.02, and 1.5% ± 0.01), BMI within health behaviors and living conditions (2.8% ± 0.01, 2.9% ± 0.05, and 2.9% ± 0.01), and physician caseload within healthcare provider information (2.8% ± 0.01, 2.5% ± 0.05, and 3.2% ± 0.01).

For the different patient samples, prior ED encounters were more important in the medical than the surgical cohort (4.1% ± 0.22 vs. 2.7% ± 0.04), whereas physician caseload was more important in the surgical compared with the medical cohort (3.2% ± 0.01 vs. 2.5% ± 0.05).

## Discussion

The present study investigated whether unplanned readmissions within 30 days can be accurately predicted using predischarge information from Swiss EMR data. Adopting a two-step approach, we previously conducted a comprehensive literature review (see [11] and further information below) to identify the most relevant predictor variables, and now engaged in significant feature engineering efforts to maximize the utility of Swiss EMR data in the present study. In collaboration with a leading Swiss private hospital group, we built random forest models to predict unplanned readmissions for a hospital-wide patient sample, as well as for separate medical and surgical subsamples across eight hospitals. We achieved robust predictive performance with AUC values up to 0.78, demonstrating the feasibility of utilizing Swiss EMR data—without post-discharge information—to predict unplanned readmissions with sufficient accuracy to inform clinical decision-making.

### Interpretation of the main findings

Most previous attempts to predict unplanned readmissions have relied on administrative data that also includes information after discharge (i.e., all coded diagnoses, procedures, and DRGs). Additionally, such studies have often utilized much larger sample sizes and included external readmissions at hospitals outside the index hospitalization. However, most previous studies have typically achieved modest prediction accuracies (see, e.g., [4]). For instance, the regression-based risk-adjustment models used by CMS, which rely on the same patient populations and readmission definitions as in our study, achieved AUC values ranging from 0.64 to 0.68.

A recent meta-analysis comparing machine learning algorithms with regression approaches for predicting hospital readmissions showed that machine learning models performed slightly better than regression models (mean difference in AUC: 0.03; 95% confidence interval: 0.01–0.05) [37]. The median AUC across all machine learning studies included in a large review was 0.68 (interquartile range: 0.64–0.76), with most studies utilizing electronic medical records (56%) and tree-based methods (53%) [38]. For example, a recent large-scale study from our neighboring country, Germany, employing regression approaches, tree-based methods (random forests), ensemble methods (AdaBoost), and neural networks reported AUC values between 0.68 and 0.69 [6,7]. In comparison, we achieved prediction accuracies with AUC values ranging from 0.72 to 0.78, despite relying solely on predischarge EMR information and operating within the constraints of a real-world clinical setting. For example, as is common when working with EMR data [8,9], we encountered many missing values and went to some length to transform structured and unstructured EMR data into a format suitable for machine learning predictions.

Our robust model performance is likely due to the nuanced patient information available in EMR data [10] and the detailed feature engineering we employed based on our prior overview of systematic reviews on predictors of readmissions. More specifically, we previously conducted an overview of systematic reviews combined with a meta-analytical approach [11] to identify the most frequently used predictors of hospital readmissions across 28 systematic reviews encompassing 440 primary studies. The identified predictors were then mapped to common ontologies to examine the odds ratios of overarching predictor groups and subgroups. As part of the present study, we replicated these predictor groups and subgroups using Swiss EMR data to build our prediction models.

Beyond model development, we also determined the feature importance of our predictor variable groups and subgroups to inform future research and guide practical implementation efforts. Our current results are in line with the findings from our previous overview of systematic reviews that found diagnoses, procedures, medications, and prior use of healthcare services to be the most frequently used and most relevant predictor variables for readmissions [11,39], In particular, in agreement with prior reports [11,39], patients’ diagnoses were the most important predictors in our model. This finding aligns with clinical intuition since patients with chronic and multimorbid conditions are at higher risk for readmissions. However, in contrast to existing studies using other data sources, we found that nursing assessments (i.e., using the EPA and LEP) provided important additional information for predicting unplanned readmissions. This highlights the significance of comprehensive nursing documentation, which captures critical patient status information that may be used for predictive modelling and patient stratification, and is not otherwise reflected in structured clinical data. We found that despite the frequent use of patient demographics, admission information, and DRGs in previous readmission prediction models [11], these predictor groups contributed relatively little to our model’s decision, presumably because more relevant predictors were available.

Regarding predictor subgroups, we found that the presence of specific ICD-10 GM, CHOP, or ATC codes was the most influential information for the model. A similar situation was true for laboratory values, where the mere information of whether specific laboratory tests were performed proved more informative than the actual test results, perhaps because the choice of tests indirectly reflects clinical indications. These findings are promising for other hospitals with limited data availability that seek to employ prediction models for readmissions.

Consistent with previous research [11,40], we confirmed that prior healthcare use is a critical source of information for predicting unplanned readmissions. This includes prior hospitalizations, but more notably, prior emergency encounters. Among vital signs, blood pressure was the most relevant predictor of unplanned readmissions, while BMI was the most important predictor within the group of health behaviors and living conditions. These findings are unsurprising, because high blood pressure and obesity are well-established cardiovascular risk factors [41]. However, the magnitude of their relevance was striking given the high proportion of missing values in these variables (ranging from 30.5% to 44.8%). Our results therefore highlight areas for improvement in data collection and documentation, especially since these variables are important for other quality-related outcomes (e.g., in-hospital complications) [42] in addition to predicting unplanned readmissions.

Comparison of medical and surgical populations provided further valuable insights for hospitals with different patient samples. For instance, prior healthcare use and medications were more important in the medical cohort, while procedural information (e.g., data from surgeries, anesthesia, and artificial ventilation) and healthcare provider information proved more relevant in the surgical cohort. The respective relevance of medications and surgical procedural information for medical and surgical patients needs no further comment; However, the particular relevance of physician caseload in influencing the readmission risk for surgical patients is an interesting finding that warrants further attention. There is a well-established relationship between higher physician caseload and improved patient outcomes across many quality dimensions (see, e.g., [43,44]), yet this information has rarely been used in prediction models [11], most likely due to its limited availability in the data sources utilized.

### Clinical relevance, limitations, and conclusion

From a clinical perspective, our findings have several important implications. First, the prediction models developed here may be employed in clinical decision support systems to stratify patients according to their risk for readmission. Second, by considering the most important predictor variables (such as diagnoses, procedures, and medications), hospitals could focus on improving pre- and post-discharge follow-up care for high-risk patients. For instance, patients with multimorbid conditions or those with complex medication regimens may benefit from targeted interventions including follow-up appointments or medication reconciliation [45]. Finally, the identified opportunities for targeted improvements in documentation quality (e.g., regarding health behaviors like weight management, see, e.g., BMI above) offer the potential to further enhance prediction modeling and patient stratification.

The most significant limitation of this study concerns its generalizability. The exact prediction models developed here may not be directly applicable to different patient populations, healthcare settings, and data sources. For this reason, we have provided detailed information on various patient samples and predictor variable groups to enable healthcare providers facing different circumstances to build on our results (i.e., depending on their available data). From a methodological perspective, it is important to note that the Gini feature importance used to compare predictor relevance tends to overestimate the importance of variables with many categories and continuous variables. Additionally, missing values may have biased the results—particularly for certain predictor variables—even though imputation strategies were applied to mitigate this issue. Finally, while our results indicate associations between predictor variables and unplanned readmissions, they do not necessarily imply causal relationships. Further research is warranted to replicate our findings in different settings with different methods and to confirm causal relationships that could be leveraged to improve patient outcomes.

In conclusion, we have demonstrated that Swiss EMR data available before discharge can be used to accurately predict unplanned readmissions within 30 days. We built robust machine learning models that achieved an average AUC value of 0.78 ± 0.01 (mean + standard deviation [SD]). Our findings on the importance of different variable groups and subgroups may be used to guide future research and support improvements in clinical practice. Our results show that predicting unplanned readmissions based on Swiss EMR data is feasible and could serve as a foundation for clinical decision support systems that target high-risk patients before discharge. This could improve patient outcomes, reduce healthcare expenditures, and ultimately contribute to enhanced quality of care.

## Supporting information

S1 Appendix Comparison of the original CMS method of identifying unplanned readmissions with our Swiss-adapted version. (DOCX)

S2 Fig Individual ROC curves across the train–test splits of the HWR patient population. (TIF)

S3 Fig Individual ROC curves across the train–test splits of the MED patient population. (TIF)

S4 Fig Individual ROC curves across the train–test splits of the SURG patient population. (TIF)

S5 Fig Average ROC curves across the train–test splits and patient populations comparing the random forest (RF) and XGBoost (XGB) models. (TIF)
