# Supplementary material for: Machine learning predictions of unplanned readmissions using electronic medical records: Predictor importance across medical and surgical patient populations
Source: PLoS One. 2025 Sep 4;20(9):e0331263. doi: 10.1371/journal.pone.0331263 (PMC12410744; doi:10.1371/journal.pone.0331263)
Supplement: S1 Appendix — (DOCX) [file pone.0331263.s001.docx]

S1 Appendix: Comparison of the original CMS method of identifying unplanned readmissions with our Swiss-adapted version.

The CMS method distinguishes between “planned” and “unplanned” 30-day readmissions. Diagnosis and procedure codes from the coded medical data of hospitals are used to identify readmissions with diagnoses or procedures that are judged as planned (e.g., organ transplants). Readmissions with such diagnoses or procedures are classified as planned readmissions. The remaining readmissions are checked for procedures that could have potentially been planned. If there are no potentially planned procedures, the readmission is judged as unplanned. If potentially planned procedures are identified, the algorithm searches for diagnosis codes that are acute or correspond to complications of care. If acute diagnoses or complications are identified, the readmission is judged as unplanned; otherwise, it is regarded as planned. The CMS method is described and illustrated in the cited publication by Horwitz et al. [12].

Our adapted version of the CMS method was translated into the Swiss medical coding system and works similarly to the original version of the CMS method, with one exception: In addition to the information for the distinction of unplanned vs. planned readmissions taken from the diagnosis and procedure codes, the hospitals’ own (routinely coded) assessment of whether readmissions were emergent or elective is also used to improve the distinction between planned and unplanned readmissions. More specifically, in readmissions without diagnosis or procedure codes that are considered as planned or potentially planned according to the original CMS method, readmissions are only classified as unplanned if this assessment is consistent with the hospitals’ assessment of whether readmissions were emergent or elective. It must be noted, though, that this additional information on the urgency of readmissions is only used to distinguish between unplanned and planned readmissions in the proportion of readmissions without any planned or potentially planned diagnosis or procedure codes. Otherwise, the adapted version of the CMS method relies on the same algorithm as the original CMS method to distinguish unplanned from planned readmissions. Therefore, the determination by the original CMS method (based on diagnosis and procedure codes) takes precedence over the hospital’s own assessment.

Furthermore, the hospitals’ assessment of the urgency of readmissions is only available for readmissions occurring after 18 days. This is because in Switzerland, readmissions within this time frame are reimbursed and reported together with the index hospitalization if they occur in the same hospital and have the same major diagnostic category (MDC). The two combined stays can be subsequently redivided into two separate stays to identify the case characteristics that are required to characterize readmissions (e.g., the dates of the intermediate discharge and the readmission, the procedure codes with their treatment dates, etc.). However, certain administrative variables (such as the distinction between emergent vs. elective readmissions) are not present in these readmissions that are reimbursed and reported together with the index admission. Thus, in these instances, no information on the urgency of the readmission is contained within the Swiss hospital administrative data, and the algorithm used in the adapted version of the CMS method is identical to the original CMS method.
