# Supplementary figures and images for: Machine learning predictions of unplanned readmissions using electronic medical records: Predictor importance across medical and surgical patient populations
Source: PLoS One. 2025 Sep 4;20(9):e0331263. doi: 10.1371/journal.pone.0331263 (PMC12410744; doi:10.1371/journal.pone.0331263)

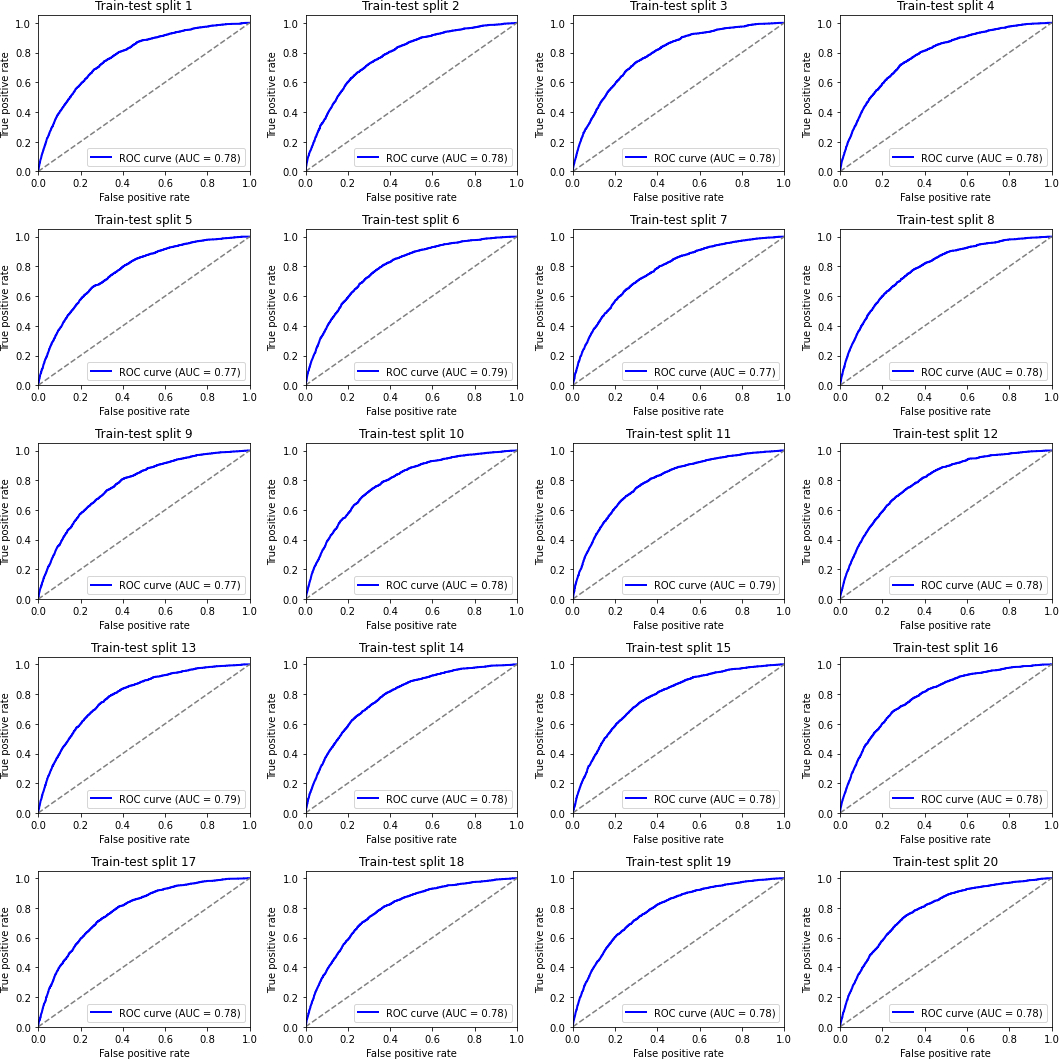

Supplement: S2 Fig — (TIF) [file pone.0331263.s002.tif]

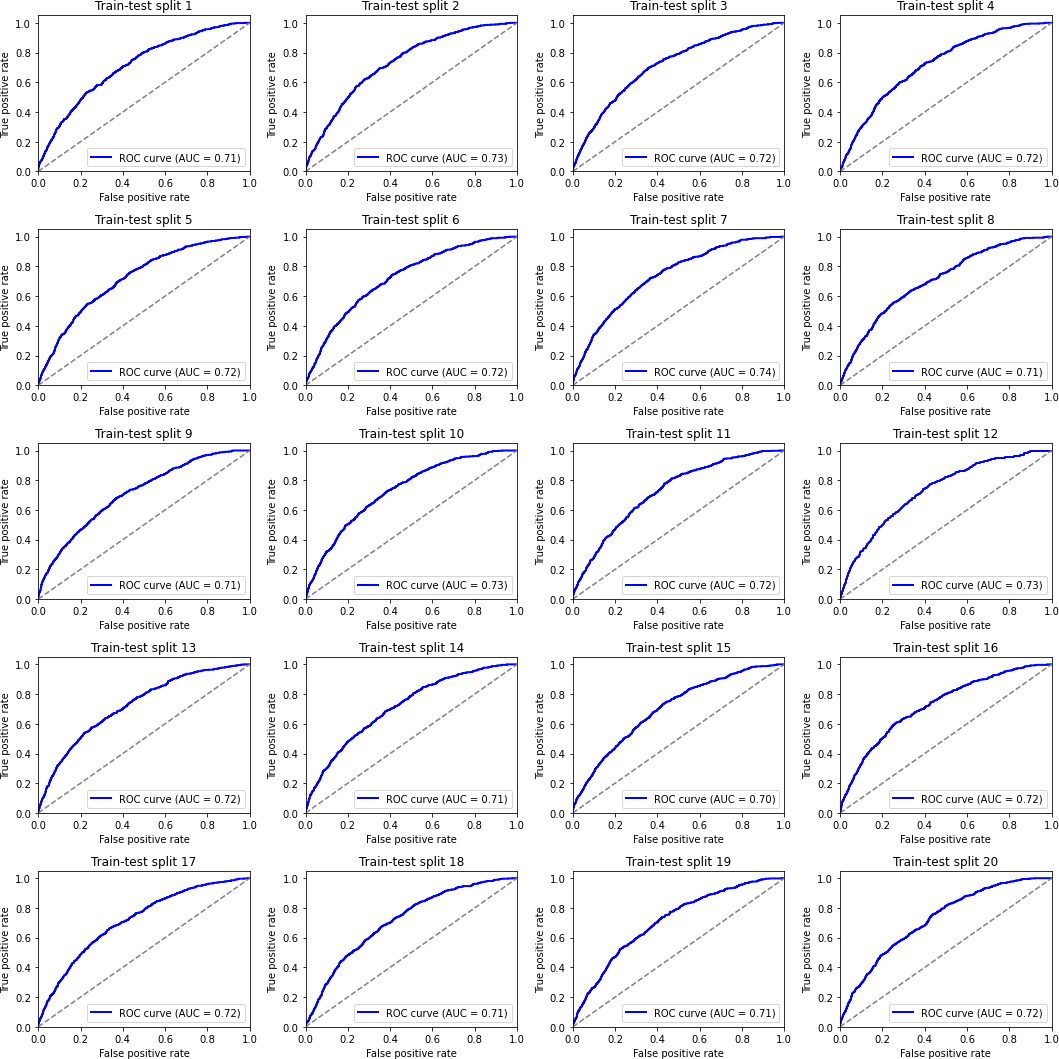

Supplement: S3 Fig — (TIF) [file pone.0331263.s003.tif]

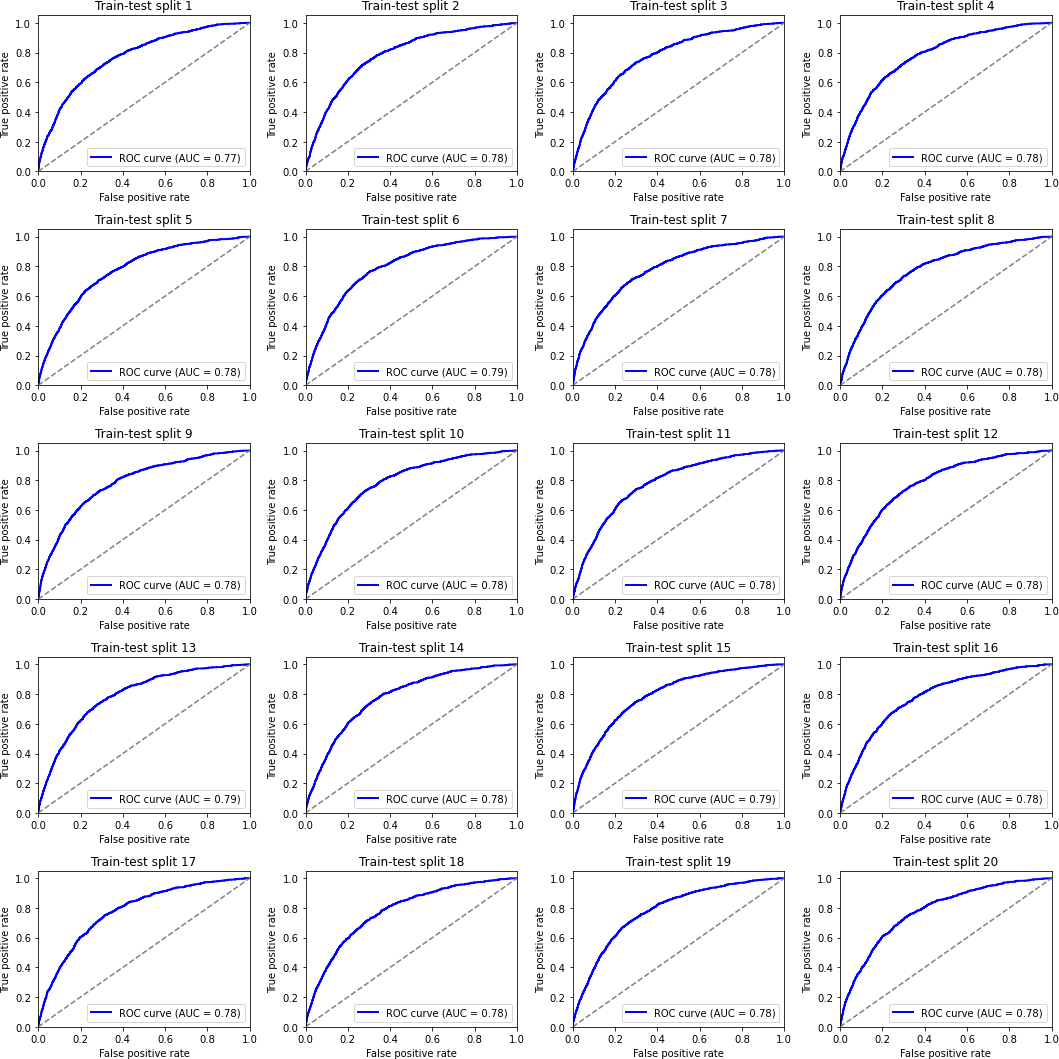

Supplement: S4 Fig — (TIF) [file pone.0331263.s004.tif]

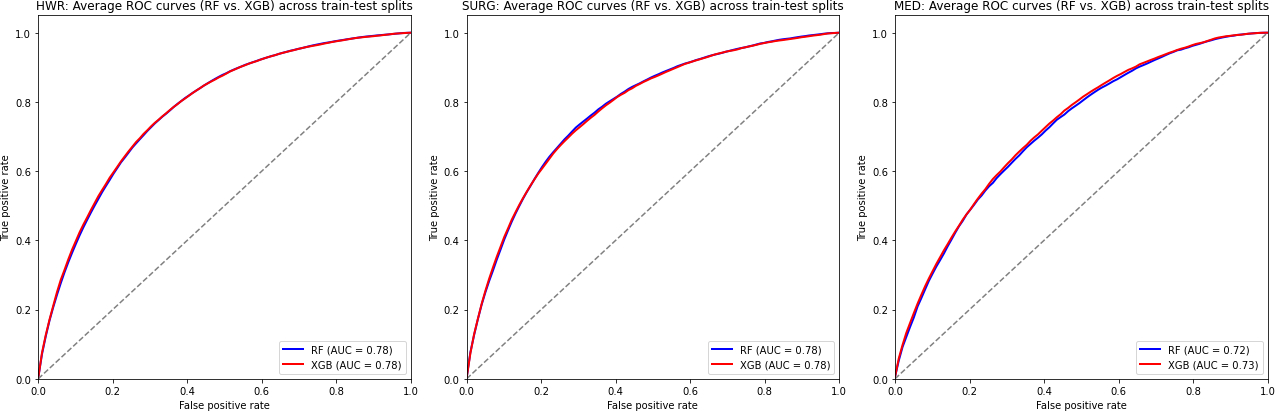

Supplement: S5 Fig — (TIF) [file pone.0331263.s005.tif]
